# New Sources of Eastern Filbert Blight Resistance and Simple Sequence Repeat Markers on Linkage Group 6 in Hazelnut (*Corylus avellana* L.)

**DOI:** 10.3389/fpls.2021.684122

**Published:** 2021-06-14

**Authors:** Golnaz Komaei Koma, Merve Şekerli, Jacob W. Snelling, Shawn A. Mehlenbacher

**Affiliations:** Department of Horticulture, Oregon State University, Corvallis, OR, United States

**Keywords:** *Anisogramma anomala*, *Corylus avellana*, eastern filbert blight, hazelnut, simple sequence repeat, microsatellite, disease resistance, linkage map

## Abstract

Commercial production of hazelnut (*Corylus avellana*) in Oregon’s Willamette Valley is threatened by eastern filbert blight (EFB), a serious canker disease caused by the pyrenomycete *Anisogramma anomala* (Peck) E. Müller. The fungus also prevents the establishment of hazelnut orchards in eastern North America. Genetic resistance is considered the most effective way to control the disease. A high level of EFB resistance was first discovered in ’Gasaway’. This resistance is conferred by a dominant allele at a single locus on linkage group 6 (LG6). Resistance from several additional sources has been assigned to the same chromosomal region. In this study, new simple sequence repeat (SSR) markers were developed for the resistance region on LG6 and new sources of resistance were investigated. Forty-two new SSR markers were developed from four contigs in the genome sequence of ‘Jefferson’ hazelnut, characterized, and nine of them were placed on LG6 of the genetic map. Accessions representing 12 new sources of EFB resistance were crossed with susceptible selections resulting in 18 seedling populations. Segregation ratios in the seedling populations fit the expected 1:1 ratio for 10 sources, while one source showed an excess of resistant seedlings and another showed an excess of susceptible seedlings. Based on correlation of disease response and scores of SSR markers in the ‘Gasaway’ resistance region in the seedlings, eight resistance sources were assigned to LG6. Linkage maps were constructed for each progeny using SSR markers. The LG6 resistance sources include two selections (#23 and #26) from the Russian Research Institute of Forestry and Mechanization near Moscow, four selections from southern Russia, one selection (OSU 1185.126) from Crimea, one selection (OSU 533.129) from Michigan, *Corylus heterophylla* ‘Ogyoo’ from the South Korea, and the interspecific hybrid ’Estrella #1’. These new LG6 resistance sources and SSR markers should be useful in breeding new cultivars, including the pyramiding of resistance genes. For the other four resistance sources (Moscow #37, hybrid selection OSU 401.014, *C. americana* ‘Winkler’ and *C. americana* OSU 366.060), SSR marker scores on linkage groups 6, 7 and 2 were not correlated with disease response and merit further investigation.

## Introduction

Hazelnut is an important tree nut. Cultivars of European hazelnut (*Corylus avellana*), also known as filbert, are clonally propagated, highly heterozygous, monoecious, dichogamous, wind-pollinated and diploid with 11 pairs of chromosomes (2*n* = 2x = 22). Traditional propagation is by the rooted suckers that grow around the crown of the plant. Nearly all of the world’s hazelnuts are of the European species, *Corylus avellana*, which is a member of the family Betulaceae. The European hazelnut is widely distributed in Europe, Turkey and the Caucasus republics, but commercial production is limited to areas near large bodies of water at middle latitudes with moderate temperatures in winter and summer, and high humidity during mid-winter bloom (Mehlenbacher, 1991). These areas include the Black Sea coasts of Turkey and Georgia, areas in Italy and Spain near the Mediterranean Sea, southwestern France near the Bay of Biscay, and the Willamette Valley of Oregon, United States near the Pacific Ocean. Turkey produces 67% of the world crop, followed by Italy, Azerbaijan, and the United States^1^.

Simple sequence repeat (SSR) markers, also known as microsatellites, are short tandem repeats widely distributed throughout plant genomes. They are the marker type of choice for many studies due to their ease of amplification by the polymerase chain reaction (PCR), high level of polymorphism, experimental reproducibility, ease of sharing among labs, usefulness in many progenies, and transferability among related species (Vieira et al., 2016). The steps in developing SSR markers are identifying the desired type of repeat in nucleotide sequences, designing primers complementary to the regions flanking the SSR, amplification by PCR, separating the PCR products by electrophoresis, and detecting polymorphism among individuals (Mason, 2015). Next Generation Sequencing technology allows rapid and inexpensive SSR marker development from genome and transcriptome sequences (Tang et al., 2008; Hoffman and Nichols, 2011; Vukosavljev et al., 2015; Bhattarai and Mehlenbacher, 2017, 2018; Colburn et al., 2017; Khodaeiaminjan et al., 2018; Taheri et al., 2018). Post-PCR multiplexing of products reduces the cost of allele sizing by capillary electrophoresis. Akın et al. (2016) identified a set of 14 primer pairs for pre-PCR multiplexing, further simplifying and reducing the cost of the procedure. More than 900 polymorphic SSR markers have been developed in *C. avellana* (Bassil et al., 2005a,b, 2013; Boccacci et al., 2005, 2015; Gürcan and Mehlenbacher, 2010a,b; Gürcan et al., 2010; Bhattarai and Mehlenbacher, 2017, 2018; Colburn et al., 2017; Öztürk et al., 2017; Zhao et al., 2019; Kavas et al., 2020; Hill et al., 2021; Şekerli et al., 2021) of which ∼450 have been placed on the reference linkage map (Mehlenbacher et al., 2006; Mehlenbacher and Bhattarai, 2018).

Eastern filbert blight (EFB), caused by the pyrenomycete *Anisogramma anomala*, has prevented the establishment of commercial orchards in eastern North America (Thompson et al., 1996; Capik and Molnar, 2012) and is now present throughout the Willamette Valley where 99% of the United States hazelnut crop is produced^2^. The pathogen is native to eastern North America where it is found on the wild American hazelnut, *C. americana*, on which it causes only limited damage (Capik and Molnar, 2012). On most *C. avellana* cultivars, however, it causes large, perennial stem cankers, branch die-back, and eventual tree death after several years. The disease life cycle is now well-understood (Pinkerton et al., 1992, 1995, 1998, 2001; Stone et al., 1992; Johnson et al., 1994, 1996). The fungus is an obligate biotroph with a 2-year life cycle^3^. Ascospores are released in winter during periods of branch wetness and dispersed by rain and air currents. Hyphae from germinating spores penetrate young growing shoots in the spring, and then spread in the cambium and phloem. Cankers become visible about 15 months after infection. Controlling the disease with scouting, pruning infected branches 30 to 90 cm below the cankers, and fungicide applications is costly and labor-intensive. Alternative disease management strategies are desirable and host genetic resistance is considered the most cost-effective method (Mehlenbacher, 1995). The high level of EFB resistance first discovered in ‘Gasaway’ (Cameron, 1976) was shown to be controlled by a dominant allele at a single locus (Mehlenbacher et al., 1991). Random amplified polymorphic DNA (RAPD) markers linked to resistance were identified (Mehlenbacher et al., 2004) and the resistance locus was placed on the reference linkage map (Mehlenbacher et al., 2006). ‘Gasaway’ resistance has been extensively used in the hazelnut breeding program at Oregon State University (OSU), and several resistant cultivars and pollinizers have been released. Fungal isolates able to overcome ‘Gasaway’ resistance were recently reported in New Jersey (Molnar et al., 2010; Muehlbauer et al., 2018; Dunlevy et al., 2019). There is an urgent need to find new sources of resistance and use them in breeding. Previously studied resistance sources include OSU 408.040 from Minnesota, ‘Culpla’ from Spain, OSU 495.072 from southern Russia, and ‘Crvenje’ and ‘Uebov’ from Serbia. Resistance from all five of these sources was placed on linkage group 6 (LG6) in the ‘Gasaway’ resistance region (Sathuvalli et al., 2012; Colburn et al., 2015; Bhattarai et al., 2017b). On the other hand, resistance in ‘Ratoli’ from Spain, *C. americana* ‘Rush’ from Pennsylvania and interspecific hybrid ‘Yoder #5’ from Ohio was assigned to a region on LG7 (Sathuvalli et al., 2011a; Bhattarai et al., 2017a). Resistance in selection OSU 759.010 from the Republic of Georgia and Rutgers University selection H3R07P25 from southern Russia was assigned to a region on LG2 (Sathuvalli et al., 2011b; Honig et al., 2019). Additional sources of EFB resistance have been identified, including Moscow #26 (Sathuvalli et al., 2010), *C. heterophylla* ‘Ogyoo’ (Coyne et al., 1998), and interspecific hybrid ‘Estrella #1’ (Chen et al., 2007), as well as germplasm collected in Russia, Crimea and Poland (Molnar et al., 2007; Capik et al., 2013; Leadbetter et al., 2016). Muehlbauer et al. (2014) characterized a large collection of EFB-resistant selections at Rutgers University.

The goals of this study were to develop and characterize new SSR markers in the resistance region on LG6 and study EFB resistance from 12 new sources.

## Materials and Methods

### Plant Material

For characterization of the new SSR markers, a diversity panel of 50 hazelnut accessions (Table 1) including the parents of the reference mapping population (OSU 252.146 and OSU 414.062) was used. For the investigation of EFB resistance, 18 seedling populations segregating for resistance from 14 resistant parents representing 12 sources were created by crossing susceptible selections with them or an advanced selection carrying the same resistance (Table 2). The pedigrees are shown in Supplementary Material 1. The resistance sources include three selections (#23, #26, and #37) from the Russian Research Institute of Forestry and Mechanization near Moscow, Russia, four selections (H3R04P23, H3R04P28, H3R04P30, and H3R13P40) from Rutgers University that arose from seeds purchased in an outdoor market in the village of Holmskij, near Krasnodar, Russia, and one selection (OSU 1185.126) from seeds purchased near Simferopol, Crimea. The four Rutgers University selections from Holmskij and one OSU selection from Simferopol originated from seeds purchased on a collection trip in 2002 by Thomas Molnar, David Zaurov and Shawn Mehlenbacher. The seed lots were shared by the two institutions (Rutgers University and OSU) and the seed source ID numbers were listed by Molnar et al. (2007). The four Rutgers University selections had been inoculated with the EFB pathogen in greenhouse or field in New Jersey in 2005 and found to be resistant (Molnar et al., 2007; Capik et al., 2013). Additional sources of resistance include OSU 533.129, selected from a lot of open-pollinated seeds received from Cecil Farris in Lansing, Michigan, one cultivar (‘Ogyoo’) of *Corylus heterophylla* from the South Korea, and interspecific hybrid ’Estrella #1’. OSU 1181.002 carries resistance from ’Ogyoo’. ‘Estrella #1’ from private breeder Cecil Farris is a hybrid of a single accession of *C. sutchuenensis* (syn. *C. heterophylla* var. *sutchuenensis*) obtained from western China as the female parent and *C. avellana* ‘Holder’ as the pollen parent (Farris, 1974). The three remaining resistance sources investigated are two clones of *C. americana* (‘Winkler’ and OSU 366.060) and the interspecific hybrid OSU 401.010 that originated from open-pollinated seeds sent from New Carlisle, Ohio by Ken Bauman, a long-time member of the Northern Nut Growers Association. The phenotype of OSU 401.040 indicates that it is a *C. americana* × *C. avellana* hybrid. *C. americana* OSU 366.060, preserved as PI 433984 at the USDA National Clonal Germplasm Repository in Corvallis, OR, was selected from a seed lot received from Mississippi. Four resistant parents (Rutgers University selections H3R04P23 and H3R13P40, OSU 533.129 and ‘Estrella #1’) were each represented by two segregating progenies, and the remaining ten parents were each represented by a single progeny.

**TABLE 1:** Hazelnut accessions used for characterization of 42 new polymorphic simple sequence repeat (SSR) markers.

**No.**	**Accession**	**Inventory no.**	**Origin**	**Source**
1	OSU 495.049	PI 557421	Russia-Southern	Southern Russia (seeds)
2	Albania 55	PI 617207	Albania	Cajup, Albania (seeds)
3	Fusco Rubra	PI 557047	Germany	Morton Arboretum, Lisle, IL, United States (scions)
4	Ala Kieri (CCOR 187)	PI 557080	Finland	Lappa, Finland (seeds)
5	Pendula	PI 557048	France	Arnold Arboretum, Boston, MA, United States (scions)
6	Hall’s Giant	PI 557027	Germany/France	OSU Entomology Farm, OR, United States (scions)
7	Gasaway	PI 557042	United States-Washington	Orchard in Washington, United States (scions)
8	Rode Zeller	PI 271280	Netherlands	Q.B. Zielinski from Netherlands (scions)
9	Cosford	PI 557039	England- Reading	NYAES, Geneva, NY, United States (scions)
10	Du Chilly	PI 557099	England	OSU Entomology Farm, OR, United States (scions)
11	Palaz	PI 304632	Turkey-Ordu	Q.B. Zielinski froom Greece (scions)
12	Pellicule Rogue	PI 271110	France	Q.B. Zielinski from France (scions)
13	Imperiale de Terbizonde	PI 271105	Turkey	Q.B. Zielinski from France (scions)
14	Tombul Ghiaghli	PI 304634	Turkey	Q.B. Zielinski from Greece (scions)
15	Tonda Bianca	PI 296206	Italy Campania	Q.B. Zielinski from Italy (scions)
16	Negret	PI 270340	Spain-Tarragona	Q.B. Zielinski from Spain (scions)
17	Tonda Gentile delle Langhe	PI 557075	Italy Piemonte	Univ. di Torino, Italy (scions)
18	Tonda Romana	PI 557025	Italy Lazio	ISF Rome, Italy (scions)
19	Römische Nuss	PI 557171	unknown	Hermansverk, Norway (scions)
20	Casina	PI 557033	Spain-Asturias	Q.B. Zielinski from Asturias, Spain (scions)
21	Ratoli	PI 557167	Spain-Tarragona	IRTA Mas Bove, Reus, Spain (scions)
22	Mortarella	PI 339723	Italy Campania	Q.B. Zielinski from Italy (scions)
23	Tonda di Giffoni	PI 296207	Italy Campania	Q.B. Zielinski from Italy (scions)
24	Barcelona	PI 557156	Spain	Oregon nursery, United States (tree)
25	Cutleaf	PI 557306	England	Arnold Arboretum, Boston, MA, United States (scions)
26	OSU 681.078	PI 634203	Russia-Moscow	Moscow, Russia (seeds, J. Henkin)
27	Barcelloner Zellernuss	PI 557156	Spain?	Faversham, England, United Kingdom (scions)
28	Aurea	PI 557050	France	Morton Arboretum, Lisle, IL, United States (scions)
29	OSU 408.040	PI 617266	Univ. Minnesota	Univ. Minnesota research farm (seeds)
30	Des Anglais	PI 557423	unknown	INRA, Bordeaux, France (scions)
31	OSU 26.072	PI 323961	Russia-North Caucasus	North Caucasus, Russia (seeds, H. Brooks)
32	Bergeri	PI 557114	Belgium-Luttich	ISF Rome, Italy (scions)
33	Alli	PI 686862	Estonia	Polli, Estonia (scions)
34	Kadetten	PI 557090	Germany	Faversham, England, United Kingdom (scions)
35	OSU 759.010	–	Georgia	L. Lazareishivili, Tbilisi, Georgia (scions)
36	Contorta	PI 557049	England	Arnold Arboretum, Boston, MA, United States (scions)
37	OSU 556.027	PI 617269	Turkey-Istanbul	Istanbul market, Turkey (seeds)
38	B-3	PI 557122	Macedonia	Skopje, Macedonia (scions)
39	OSU 54.039	PI 557060	Turkey-Giresun/Ordu	Giresun, Turkey (seeds, M. Thompson)
40	Gunslebert	PI 557191	Gunsleben, Germany	INRA Bordeaux, France (scions)
41	Sant Jaume	PI 557103	Spain-Tarragona	IRTA Mas Bove, Reus, Spain (scions)
42	Iannusa Racinante	PI 557183	Italy Sicily	Univ. di Torino, Italy (scions)
43	Gem	PI 557029	Washington	Orchard in Oregon, United States (scions)
44	Artellet	PI 557108	Spain-Tarragona	IRTA Mas Bove, Reus, Spain (scions)
45	Simon	PI 557166	Spain-Tarragona	IRTA Mas Bove, Reus, Spain (scions)
46	Gustav’s Zellernuss	PI 557085	Landsberg	Faversham, England, United Kingdom (scions)
47	Buttner’s Zeller	PI 557094	Germany Landsberg	Faversham, England, United Kingdom (scions)
48	Tapparona di SCC^z^	PI 617239	Italy Liguria	Univ. di Torino, Italy (scions)
49	OSU 252.146	–	Oregon State University	parent of mapping population 93001
50	OSU 414.062	–	Oregon State University	parent of mapping population 93001

*^*z*^Tapparona di San Colombano Cortemoli. Accessions 1 to 24 were used to screen for polymorphism on agarose gels, while all 50 accessions were used for characterization of the markers. Some accessions were imported as scions and others as seed lots from which individuals were selected. The question mark (?) means that this cultivar obtained from England supposedly is of Spanish origin.*

**TABLE 2:** Segregation for eastern filbert blight response in 18 hazelnut progenies from 14 resistant parents and 12 sources following structure or field inoculation.

				**Disease response**		**Expected**	**χ^2^**	
**Progeny**	**Parentage (female × male)**	**Resistance source**	**Inoculation method**	**Resistant**	**Susceptible**	**ratio**	**Value**	***P***
12028	OSU 1266.005 × Moscow #23	Moscow #23	Structure	33	20	1:1	3.180	0.075
			Field	13	12	1:1	0.040	0.841
			Pooled data	46	32	1:1	2.510	0.113
			Heterogeneity	−	−		0.720	0.396
12029	OSU 919.031 × Moscow #26	Moscow #26	Structure	19	21	1:1	0.100	0.752
			Field	7	18	1:1	4.840	0.028
			Pooled data	26	39	1:1	2.600	0.107
			Heterogeneity	−	−		2.340	0.126
12032	Moscow #37 × OSU 1218.068	Moscow #37	Field	24	61	1:1	16.000	0.000
14027	H3R04P23 × OSU 978.058	Holmskij, Russia (RUS-13)	Structure	19	21	1:1	0.100	0.752
14028	H3R04P23 × OSU 1378.046	Holmskij, Russia (RUS-13)	Structure	36	35	1:1	0.014	0.906
			Pooled data	55	56	1:1	0.009	0.924
			Heterogeneity				0.005	0.746
14029	Sacajawea × H3R04P28	Holmskij, Russia (RUS-13)	Structure	38	23	1:1	3.688	0.055
14030	Sacajawea × H3R04P30	Holmskij, Russia (RUS-13)	Structure	28	25	1:1	0.169	0.681
14036	H3R13P40 × OSU 978.058	Holmskij, Russia (RUS-9)	Structure	29	20	1:1	1.653	0.198
14037	H3R13P40 × OSU 1078.043	Holmskij, Russia (RUS-9)	Structure	16	13	1:1	0.310	0.577
			Pooled data	45	33	1:1	1.846	0.174
			Heterogenetiy				0.576	0.161
11025	OSU 1185.126 × OSU 856.064	Simferopol, Crimea (RUS-26)	Structure	14	11	1:1	0.360	0.550
			Field	38	32	1:1	0.510	0.470
			Pooled data	52	43	1:1	0.853	0.356
			Heterogeneity				0.017	0.896
14023	OSU 1390.008 × OSU 919.031	Farris OSU 533.029	Structure	38^*z*^	16	1:1	8.962	0.002
14024	OSU 1390.008 × OSU 1322.038	Farris OSU 533.029	Structure	40	16	1:1	10.285	0.001
			Pooled data	78	32	1:1	19.236	<0.001
			Heterogeneity				0.011	<0.001
10021	OSU 1181.002 × OSU 1093.107	*C. heterophylla* ‘Ogyoo’	Structure	48	40	1:1	0.720	0.390
11520	Estrella #1 × OSU 1174.033	*C. sutchuenensis*	Structure	10	24	1:1	5.760	0.020
			Field	21	33	1:1	2.670	0.100
11521	Estrella #1 × OSU 1219.032		Structure	11	11	1:1	0.000	1.000
			Field	26	19	1:1	1.090	0.296
			Pooled data	73	81	1:1	0.416	0.519
			Heterogeneity (df = 3)	−	−		3.261	0.353
11027	OSU 1197.113 × OSU 1172.001	*C. americana* ‘Winkler’	Structure	30	30	1:1	0.000	1.000
			Field	16	20	1:1	0.440	0.500
			Pooled data	46	50	1:1	0.160	0.690
			Heterogeneity	−	−	1:1	0.280	0.600
11032	OSU 955.028 × OSU 1213.088	*C. americana* OSU 366.060	Structure	29	29	1:1	0.000	1.000
11029	OSU 889.084 × OSU 1155.009	Bauman hybrid OSU 401.014	Structure	18	14	1:1	0.500	0.480
			Field	28	20	1:1	1.330	0.250
			Pooled data	46	34	1:1	1.800	0.180
			Heterogeneity	−	−	1:1	0.030	0.860

*Ratios for the two progenies segregating for resistance from Farris OSU 533.029 fit a 3:1 ratio, but SSR markers indicated a total of 19 seedlings free of disease had escaped infection.*

### *In silico* Development of New SSR Markers in the Resistance Region

The genome sequence of ‘Jefferson’ hazelnut (V2), assembled from Pacific Biosciences (PacBio, Menlo Park, CA, United States) sequences and error-corrected using Illumina (San Diego, CA, United States) reads, was used as the reference genome for marker development. Sequences of four types predicted to be near the ‘Gasaway’ EFB resistance locus on LG6 were aligned to the reference genome using the Basic Local Alignment Search Tool (BLAST)^4^. The sequences were of RAPD markers, SSR markers, Illumina sequences of bacterial artificial chromosomes (BACs), and BAC end sequences. The identified PacBio contigs (Table 3) were investigated for presence of SSRs using the Genome-wide Microsatellite Analyzing Tool (GMATo) (Wang et al., 2013) with minimum numbers of repeats for the di-, tri-, tetra-, penta- and hexa- repeats set at 8, 6, 4, 4 and 3, respectively. Repeat motifs containing only As and Ts were not pursued as experience has shown them to be difficult to score. Samtools (Li et al., 2009) were used to trim the SSR-containing fragments, retaining the repeat motif and 250 bp on either side. One at a time, the SSR-containing fragments from ‘Jefferson’ were used as the reference and aligned *in silico* with Illumina genome sequences of seven other cultivars (’Barcelona’, ’Ratoli’, ’Tonda Gentile delle Langhe’, ’Tonda di Giffoni’, ’Daviana’, ’Hall’s Giant’, and ’Tombul’) (Rowley et al., 2018). The aligned reads were visualized using Tablet software (Milne et al., 2010), inspected, and classified as “not polymorphic”, “slightly polymorphic”, or “clearly polymorphic”. Only those in the latter category were pursued. Forward and reverse primers (Supplementary Material 3) were designed from the conserved sequences that flanked each selected SSR using Websat (Martins et al., 2009) and Primer3 software (Untergasser et al., 2012) with parameters set at annealing temperature 60°C, a minimum GC content of 50%, and an amplicon size of 90-350 bp to facilitate post-PCR multiplexing of primer products for genotyping. A BLAST search against the NCBI database was used to verify that the sequences had not previously been used for SSR development. DNA of 24 accessions (Table 1) was amplified with each pair of primers in GeneAmp PCR system 9700 thermal cyclers (Applied Biosystems, Foster City, CA, United States) in 96-well plates as follows: denaturation at 95°C for 5 min followed by 40 cycles of 94°C for 40 s, 60°C for 40 s, 72°C for 40 s, extension at 72°C for 7 min, and a final infinite hold at 4°C. Polymorphic SSRs were identified by separating the PCR products for 2.5 h at 90V on 3% w/v agarose gels in TBE buffer. The gels were stained with ethidium bromide and images were recorded under ultraviolet light using a BioDoc-It^®^ Imaging System (UVP, Upland, CA, United States).

**TABLE 3:** New simple sequence repeat markers developed from three contigs in the ‘Jefferson’ hazelnut genome sequence (V2) and the random amplified polymorphic DNA (RAPD) marker and bacterial artificial chromosome (BAC) sequences used to identify the contigs.

			**Simple sequence repeat markers (all have a GK prefix)**		
**RAPD marker**	**BAC**	**Contig**	**Mapped markers**	**Unmapped markers**	**Linkage group**
152-800	53B4	42F	1.05, 1.10, 1.12, 1.20	1.09, 1.17, 1.18, 1.21, 1.24, 1.25	1
H04-850	78A4	56F	1.30, 1.38, 1.40, 1.41, 1.44, 1.45	1.35, 1.36, 1.39, 1.43, 1.46, 1.49	1
152-800	56N1	95F	6.61, 6.63, 6.77, 6.81, 6.84, 6.89, 6.90, 6.92, 6.94	6.58, 6.62, 6.70, 6.71, 6.76, 6.80,	6
W07-375	43F13	95F		6.82, 6.83, 6.91, 6.95, 6.97	6
X01-825	96K15	95F			

### Characterizing New Polymorphic SSR Marker Loci

Fluorescent forward primers with a label of FAM or HEX (Integrated DNA Technologies, Coralville, IA, United States) were ordered for the SSRs considered “clearly polymorphic” on agarose gels. DNA from 48 hazelnut accessions and the two parents of the reference mapping population (Table 1) was amplified with the fluorescent forward and non-fluorescent reverse primers. An aliquot of 2 μL of the PCR product of each primer pair was diluted with water to make a final volume of 150 μL. An average of six primer pairs was post-PCR multiplexed in a single well. An aliquot of 1.6 μL of the multiplex was submitted to the Core Labs of OSU’s Center for Genome Research and Biocomputing (CGRB) for fragment sizing by capillary electrophoresis on an ABI 3730 (Life Technologies, Carlsbad, CA, United States) with ROX-500 as the size standard. GeneMapper software (Life Technologies) was used for allele size determination followed by manual verification. Characterization of marker loci was carried out using PowerMarker (Liu and Muse, 2005) and Cervus software (Kalinowski et al., 2007). PowerMarker software was used to calculate the number of alleles (n), observed heterozygosity (H_o_), expected heterozygosity (H_e_), and polymorphism information content (PIC) for each locus. The frequency of null alleles was calculated with Cervus software (Table 4).

**TABLE 4:** Characteristics of 42 new simple sequence repeat markers developed from four contigs in the V2 genome sequence of ‘Jefferson’ hazelnut (*Corylus avellana*).

**Marker**	**GenBank accession no.**	**Motif**	**Size**	**n**	**Ho**	**He**	**PIC**	**F(null)**	**LG**	**Contig**
GK1.05	MK913590	(TC)_9_	127	9	0.94	0.85	0.82	−0.054	1	42F
GK1.09	MT670379	(CT)_14_	124	9	0.33	0.79	0.75	0.406	1	42F
GK1.10	MK913591	(AC)_12_	290	11	0.35	0.80	0.77	0.386	1	42F
GK1.12	MK913592	(TC)_13_	127	15	0.77	0.85	0.83	0.049	1	42F
GK1.17	MT670380	(GA)_17_	124	12	0.96	0.89	0.87	−0.046	1	42F
GK1.18	MT670381	(CT)_15_	176	12	0.93	0.87	0.86	−0.042	1	42F
GK1.20	MK913593	(AG)_9_	173	9	0.75	0.74	0.70	−0.018	1	42F
GK1.21	MT670382	(GA)_10_	179	5	0.52	0.49	0.46	−0.028	1	42F
GK1.24	MT670383	(AG)_11_	310	6	0.73	0.65	0.60	−0.065	1	42F
GK1.25	MT670384	(TC)_26_	216	18	0.90	0.89	0.86	−0.013	1	42F
GK1.30	MK913594	(AG)_17_	200	8	0.85	0.80	0.76	−0.039	1	42F
GK1.35	MT670385	(CT)_18_	110	19	0.81	0.88	0.86	0.021	1	56F
GK1.36	MT670386	(CT)_13_	124	6	0.85	0.80	0.76	−0.051	1	56F
GK1.38	MK913595	(TC)_16_	135	9	1.00	0.82	0.79	−0.112	1	56F
GK1.39	MT670387	(TC)_18_	302	15	0.58	0.70	0.68	0.131	1	56F
GK1.40	MK913596	(TC)_13_	368	11	0.60	0.71	0.66	0.087	1	56F
GK1.41	MK913597	(CT)_14_	244	14	0.79	0.85	0.82	0.032	1	56F
GK1.43	MT670388	(AG)_13_	132	9	0.77	0.77	0.73	−0.002	1	56F
GK1.44	MK913598	(AG)_15_	308	8	0.75	0.74	0.69	−0.008	1	56F
GK1.45	MK913599	(TG)_12_	204	13	0.52	0.79	0.77	0.206	1	56F
GK1.46	MT670389	(CT)_16_	164	9	0.46	0.87	0.85	0.307	1	56F
GK1.49	MT670390	(GAG)_9_	110	13	0.58	0.74	0.69	0.109	1	56F
GK6.58	MT670391	(TC)_10_	319	11	0.77	0.83	0.80	0.035	6	77F
GK6.61	MK913600	(TC)_12_	110	10	0.83	0.76	0.72	−0.055	6	77F
GK6.62	MT670392	(TC)_8_	148	9	0.79	0.74	0.69	−0.048	6	77F
GK6.63	MK913601	(GA)_18_	102	11	0.96	0.87	0.84	−0.058	6	77F
GK6.70	MT670393	(GA)_17_	184	8	0.85	0.70	0.65	−0.121	6	77F
GK6.71	MT670394	(GA)_13_	97	9	0.90	0.85	0.82	−0.036	6	77F
GK6.76	MT670395	(TC)_8_	155	4	0.27	0.67	0.61	0.415	6	95F
GK6.77	MK913602	(AG)_12_	177	6	0.77	0.73	0.68	−0.032	6	95F
GK6.80	MT670396	(CT)_15_	185	14	0.83	0.86	0.84	0.011	6	95F
GK6.81	MK913603	(GA)_13_	102	9	1.00	0.84	0.81	−0.099	6	95F
GK6.82	MT670397	(TC)_15_	206	10	0.98	0.83	0.80	−0.090	6	95F
GK6.83	MT670398	(CT)_16_	256	17	0.45	0.82	0.80	0.294	6	95F
GK6.84	MK913604	(TC)_15_	296	8	0.24	0.28	0.71	0.473	6	95F
GK6.89	MK913605	(AC)_12_	235	18	0.88	0.91	0.89	0.011	6	95F
GK6.90	MK913606	(TC)_12_	191	11	0.90	0.84	0.81	−0.040	6	95F
GK6.91	MT670399	(TC)_8_	128	6	0.75	0.69	0.64	−0.055	6	95F
GK6.92	MK913607	(TC)_15_	196	10	0.83	0.78	0.74	−0.045	6	95F
GK6.94	MK913608	(GA)_12_	170	13	1.00	0.86	0.83	−0.087	6	95F
GK6.95	MT670400	(AG)_12_	191	10	0.75	0.78	0.74	0.006	6	95F
GK6.97	MT670401	(TCTT)_6_	266	17	0.44	0.77	0.74	0.301	6	95F
Mean				10.74	0.737	0.778	0.756	0.049		

*Size (bp) in V2 genome sequence, n number of alleles, Ho observed heterozygosity, He expected heterozygosity, PIC polymorphism information content, F(null) frequency of null alleles, LG linkage group.*

### Mapping New SSR Markers

To place the new SSR markers on the reference linkage map, the two-way pseudotestcross approach (Grattapaglia and Sederoff, 1994) and all 138 seedlings in the reference mapping population (OSU 252.146 × OSU 414.062) were used. Each allele size was scored as present or absent in each seedling. For analysis with Join Map 4.0 (Van Ooijen, 2006) and the BC1 function, marker present was scored as “h”, marker absent was scored as “a”, and unknown status was scored as “u”. Scores for previously mapped markers and the new markers were combined and grouped at a LOD score of 12. Linkage maps were constructed using the maximum likelihood algorithm and distances shown in Haldane units (cM). “Dummy variables” were created to allow the merger of markers linked in coupling and repulsion.

### Disease Inoculation

Two approaches were used for disease inoculation: exposure of potted trees under an inoculation structure and planting seedlings in a field near a heavily diseased orchard. Potted seedlings grown in 5 L pots were inoculated with EFB by placing them under a structure topped with diseased branches in the spring, based on Pinkerton et al. (1993). The structure was located at the OSU Smith Horticulture Research Farm in Corvallis, OR, United States. Sprinklers on top of the structure kept the inoculum branches wet, allowing spores to drip down on the seedling trees shortly after leaf budbreak. The trees were lined out in a nursery row after exposure and scored for disease response 18 months after inoculation and again 12 months later. Rooted layers of check cultivars ‘Ennis’ (highly susceptible), ‘Lewis’ (moderately resistant), and ‘Tonda di Giffoni’ (high quantitative resistance) were grown in pots and included as controls in the inoculation. Structure-inoculated seedlings were observed for the presence of cankers and stromata, and disease severity was rated on a scale of 0 to 5, with a rating of 0 for absence of disease symptoms, 1 for presence of small sunken cankers without any stromata, 2 for presence of small cankers with few stromata, 3 for presence of cankers with mature stromata, 4 for cankers all over the tree but the tree was still alive, and 5 for cankers all over the tree and the top of the tree was dead. For the initial analysis of segregation for disease response following structure exposure, seedlings with an EFB score of 0, 1, and 2 were considered resistant and seedlings with a score of 3, 4, or 5 were considered susceptible. In the second method, seedlings were planted in the field adjacent to a highly diseased orchard. Each seedling was inspected annually in the winter for 4 years (2014-2017) and presence of EFB cankers was noted. EFB-susceptible seedlings in the plot adjacent to the infected orchard, susceptible seedlings in nearby plots and susceptible selections in a replicated trial 200 m to the northwest of the infected orchard were also inspected for EFB. The presence of dwarf seedlings was also noted in the field plot. Field-exposed seedlings were scored as resistant (no cankers with stromata) or susceptible (cankers with stromata). Chi-square tests were used to assess goodness-of-fit to the 1:1 segregation ratio expected for segregation at a single locus and a heterozygous resistant parent. Heterogeneity Chi-square tests were performed when the disease response of seedlings from the same progeny were investigated by structure and field exposure to determine if the data from the two methods could be pooled. Heterogeneity Chi-square tests were also performed when two progenies segregated for resistance from the same parent to determine if the data could be pooled.

### DNA Extraction

Leaves were collected from trees growing in the field in two locations in Corvallis, OR [National Clonal Germplasm Repository of the United States Dept. of Agriculture-Agricultural Research Service and OSU’s Smith Horticultural Research Farm]. Young leaves were collected from the 50 accessions in the diversity panel (Table 1). For the progenies segregating for disease response following structure exposure, leaves were collected from seedlings in a nursery row 2 years after exposure and 4 years after the controlled cross had been made. For the progenies exposed in the field, leaves were collected in spring 2016 from progenies 10021, 11025, 11027, 11029, 11032, 11520, 11521, 12028, 12029, and 12032. Genomic DNA was extracted based on Lunde et al. (2000) with no RNAase treatment. A Synergy2 microplate reader and Gen5 software (Biotek Instruments, Winooski, VT, United States) were used to quantify the extracted DNA. The DNA was diluted with TE buffer to a concentration of 20 ng μL^–1^.

### Amplification and Scoring of SSR Markers

Polymerase chain reactions were performed in 10 μl final volumes with a mixture of 20 ng DNA, 1 × Biolase NH_4_ reaction buffer, 2.5 mM mix dNTP, 2 mM MgCl_2_, 0.3 μL of each forward and reverse primer (10 μM), and 0.25 units of Biolase DNA polymerase (Bioline USA Inc., Taunton, MA, United States). PCRs were in 96-well plates on GeneAmp PCR System 9700 thermal cyclers (Applied Biosystems, Foster City, CA, United States). The amplification program consisted of an initial denaturation for 5 min at 94°C followed by 40 cycles of 40 s at 94°C, 40 s at the annealing temperature (60 or 62°C), 40 s for elongation at 72°C, and a final extension step of 7 min at 72°C, then an infinite hold at 4°C. After PCR, products were multiplexed by mixing 2 μL from each product and diluted with water to make a final volume of 200 μL. A 1.8 μL aliquot was submitted to the Core Labs of OSU’s Center for Genome Research and Biocomputing (CGRB) for fragment sizing by capillary electrophoresis on an ABI 3730 instrument using ROX-500 as the size standard. Allele sizes were visualized and scored with ABI GeneMapper software (Life Technologies, Carlsbad, CA, United States). If amplification failed or the result was unclear, the PCR amplification and fragment sizing were repeated. Allele sizes at each SSR marker and disease scores for each seedling in each progeny were entered in a spreadsheet.

### Correlation of Disease Response and SSR Marker Scores

Three sets of SSR markers, one on LG6, a second set on LG2 and a third set on LG7 near previously mapped resistance loci were used to score the seedlings and their parents. When two progenies were available for the same resistance source, only one progeny was used for the correlation analysis. For 8 progenies (10021, 11025, 11027, 11029, 11032, 11520, 12028, and 12029), the markers were scored in 32 seedlings in the initial correlation analysis, and for the remaining five progenies (14023, 14028, 14029, 14030, and 14036), 46 seedlings were scored for the correlation analysis. In this analysis, the presence of an SSR allele or resistance was scored as 1 and absence or susceptibility was scored as 0. Pearson product-moment correlation coefficients were calculated. Coefficients ≥0.5 were interpreted as indicating linkage of disease response and the marker, while those <0.5 were interpreted as showing independence of disease response and marker scores.

### Mapping SSR Marker and Resistance Loci

Join Map 4.0 (Van Ooijen, 2006) and the BC1 function were used to construct maps for each source of resistance using the procedures described earlier for placing the new SSR markers on the reference linkage map. Resistance and presence of a marker allele were scored as “h”, susceptible and marker allele absent were scored as “a”, and unknown status was scored as “u”. Markers were grouped at a LOD score of 12 and linkage maps constructed using the maximum likelihood algorithm. Two progenies were available for each of three resistance sources (H3R04P23, H3R13P40, and OSU 533.129). Separate maps were made for each progeny in these pairs, and then the data merged and a new map constructed for that resistant parent.

### Conflicts of Disease Response and Marker Scores

Data were recorded in a spreadsheet, with presence of an SSR allele or disease resistance scored as “1” and absence or susceptibility scored as “0”. Scores for each marker and disease resistance were in separate columns, and the scores for a seedling were in a single row. The columns were then placed in order according to their positions on the reference linkage map. The SSR and disease resistance scores were inspected and individuals showing a conflict between disease score and adjacent SSR marker scores were identified. Disease scores for these seedlings were reentered as “u” for unknown, and new maps were created.

## Results

### Identification, Characterization, and Mapping of New SSRs

**FIGURE 1:**
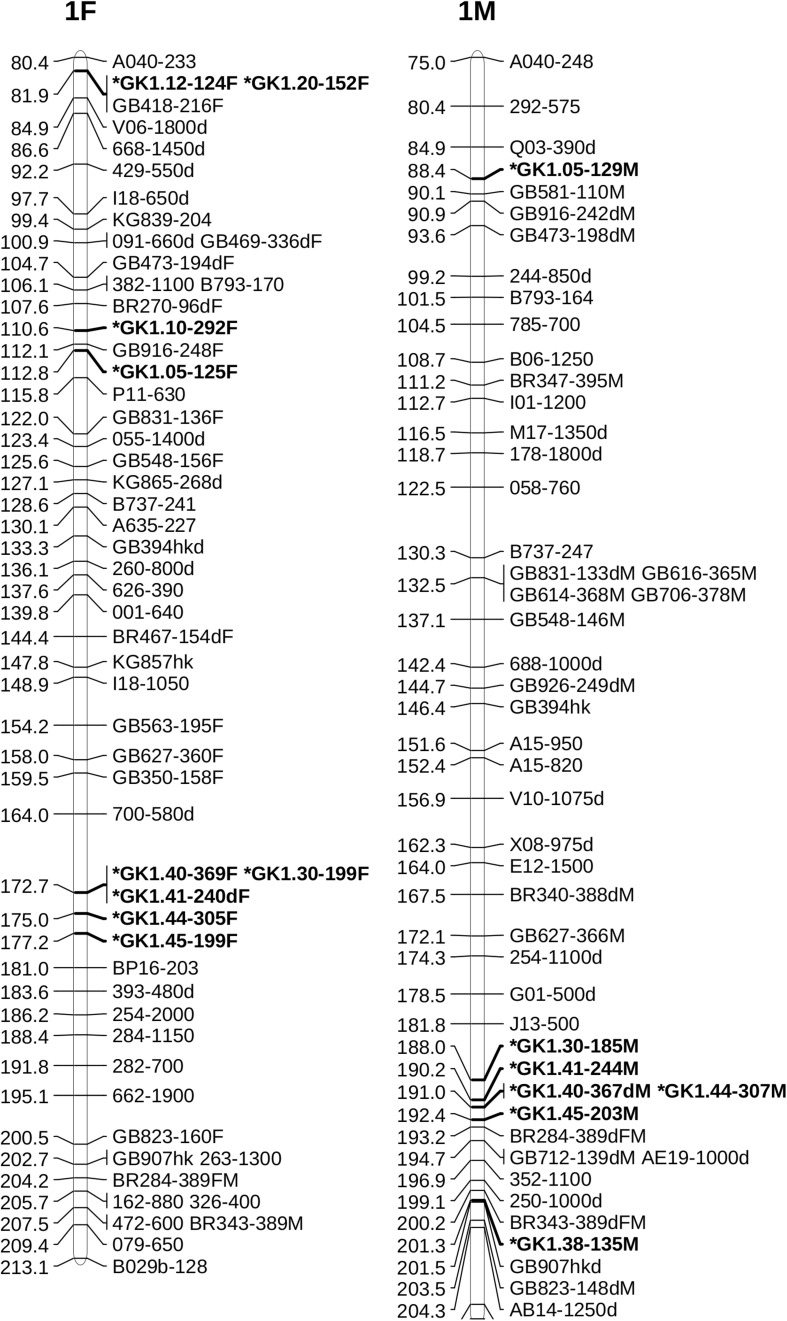
Maps of linkage group 1 (LG1) of female parent OSU 252.146 **(left)** and resistant parent OSU 414.062 **(right)** in the reference mapping population for hazelnut (*Corylus avellana*) with new simple sequence repeat markers indicated by *. Units are centimorgans (cM).

**FIGURE 2:**
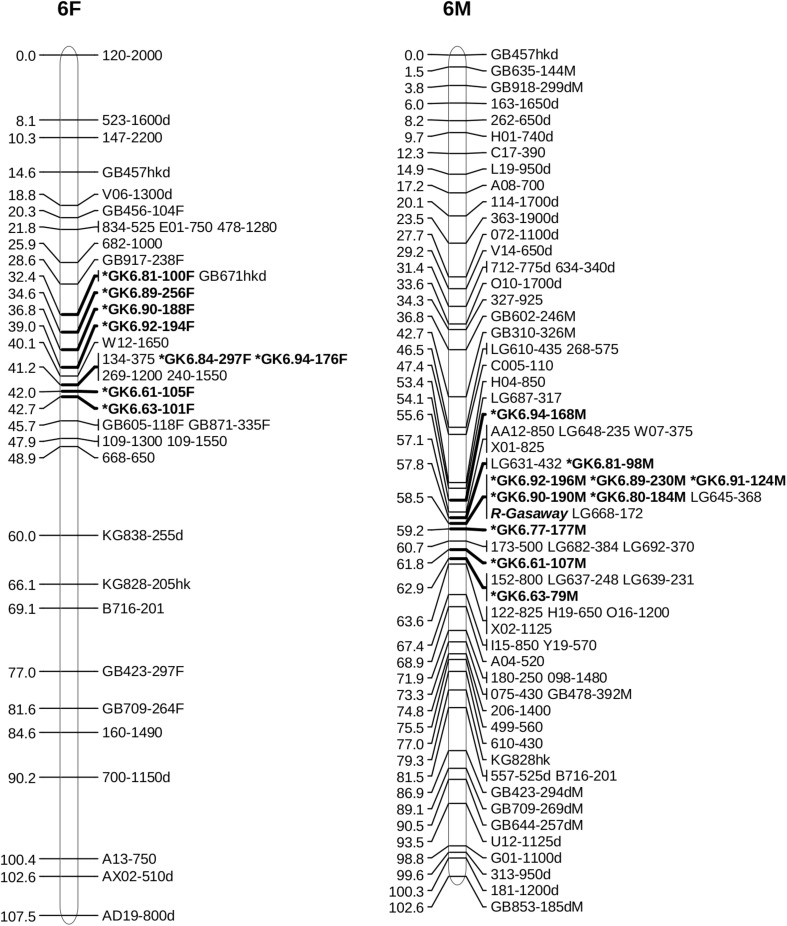
Maps of linkage group 6 (LG6) of female parent OSU 252.146 **(left)** and resistant parent OSU 414.062 **(right)** in the reference mapping population for hazelnut (*Corylus avellana*) with new simple sequence repeat markers indicated by *. Units are centimorgans (cM).

Alignment of the sequences of mapped SSR and RAPD markers, BAC end sequences, and Illumina sequences of BACs with the V2 reference ‘Jefferson’ genome identified four contigs (95F, 56F, 42F, and 77F) as being in the ‘Gasaway’ resistance region on LG6 (Table 3). A search of these four contigs for SSRs identified 896 di-, 187 tri-, 206 tetra-, 70 penta-, and 282 hexa-nucleotide repeats, with di-nucleotide repeats more abundant that the longer motifs. Removal of the repeats that contained only As and Ts reduced the number of unique fragments to 451. When the Illumina genome sequence reads of the 7 other cultivars were aligned with the trimmed ‘Jefferson’ fragments using Tablet software, 116 were identified as clearly polymorphic with variation in number of repeats but conserved flanking sequences. The alignments showed that the di-nucleotide repeats were more polymorphic than the tri-, tetra-, penta- and hexa-nucleotide repeats. After PCR amplification of 24 accessions and electrophoresis on 3% agarose gels, 60 of the 116 were scored as clearly polymorphic. A BLAST search against the NCBI database confirmed that all of the markers were different from previously developed SSR markers. Following amplification using fluorescent forward primers, allele sizes were scored in the 50 hazelnut accessions. Of the 60 markers scored as clearly polymorphic on agarose gels, 42 were easy to score while 18 were difficult to score and not pursued further. These 42 markers were characterized using 50 hazelnut accessions (Table 4). The number of alleles per locus ranged from 5 to 19 with an average of 10.7. The mean values for Ho, He and PIC were 0.74, 0.78, and 0.76, respectively. The frequency of null alleles at the 42 SSR markers averaged 0.049 and showed a range from −0.121 to 0.473. A null allele is defined as any allele that consistently fails to amplify due to primer template mismatch. The frequency of null alleles exceeded 0.20 at 8 markers (GK1.09, GK1.10, GK1.45, GK1.46, GK6.76, GK6.83, GK6.84, and GK6.97). Of the 42 markers, 20 segregated in the mapping population and were placed on the reference linkage map (Figures 1, 2). The map locations of the remaining markers were deduced based on the V2 contig from which they were developed and their coordinates in the V3 ‘Jefferson’ genome (Supplementary Material 3). Of the 20 mapped markers, nine were placed on LG1 and 11 were placed on LG6. Of the nine markers on LG1, six (GK1.05, GK1.30, GK1.40, GK1.41, GK1.44, and GK1.45) were placed on the maps of both the female and male parents and three markers (GK1.12, GK1.10, GK1.20) were placed only on the map of the female parent (Figure 1). Of the 11 markers on LG6, seven (GK6.61, GK6.63, GK6.81, GK6.89, GK6.90, GK6.92, and GK6.94) were placed on the maps of both the female and male parents, while one (LG6.84) was placed only on the map of the female parent and three (GK6.77, GK6.80, and GK6.91) were placed only on the map of the male parent (Figure 2). All of the markers assigned to LG1 were developed from contigs 56F and 42F, which had been identified using sequences of BACs 53B4 and 78A4 in the physical map of the eastern filbert blight resistance region (Sathuvalli et al., 2017). None of the markers developed from contigs 56F and 42F were placed on LG 6.

### Segregation for EFB Resistance and Linkage Group Assignment

Segregation for disease response was studied in 18 progenies representing 14 resistant parents and 12 resistance sources (Table 2). Of the 14 parents, 12 fit the 1:1 ratio expected when resistance is dominant and under the control of a single locus. The two progenies segregating for resistance from Farris OSU 533.029 had an excess of resistant seedlings, and the heterogeneity Chi-square values indicated it was appropriate to pool the data. A single progeny segregating for resistance from ‘Moscow #37’ had an excess of susceptible seedlings. The field-grown progenies segregating for resistance from ‘Estrella #1’ included several very weak seedlings with no disease, 9 in progeny 11520 and 17 in progeny 11521, of which 7 and 4, respectively, died before DNA could be extracted. After the very week seedlings were removed, the Estrella #1 progenies in the EFB nursery and field fit the expected 1:1 ratio. In six progenies, some seedlings were structure-inoculated and others were field-inoculated; in all cases the heterogeneity chi-square indicated it was appropriate to pool the results of the two inoculation methods. For the RUS-13 (Holmskij) source, heterogeneity Chi-square values indicated that it was appropriate to pool the data of the two progenies of H3R04P23, and also to pool all four progenies with RUS-13 resistance.

Correlation coefficients for disease reaction and marker scores are presented with markers grouped by LG (Tables 5, 6). In 10 progenies, disease response was highly correlated (*r* > 0.70) with allele scores for markers on LG6, and thus EFB resistance was assigned to LG6 (Table 5). In progeny 12032, which segregated for resistance from Moscow #37, there was a surplus of susceptible seedlings. In progeny 11027 segregating for resistance from *C. americana* ’Winkler’, progeny 11032 segregating for resistance from *C. americana* OSU 366.060, and progeny 11029 segregating for resistance from Bauman hybrid OSU 401.014, the data fit the expected 1:1 ratio. In these four progenies, several markers on LG6, LG2, and LG7 were tested but disease response was not correlated with any marker allele scores, and EFB resistance could not be assigned to LG6, LG2, or LG7 (Table 6).

**TABLE 5:** Correlation coefficients for disease and simple sequence repeat marker scores in ten hazelnut progenies segregating for resistance to eastern filbert blight.

**Progeny**	**12028**	**12029**	**14028**	**14029**	**14030**	**14036**	**11025**	**14023**	**10021**	**11520**

**Resistance source**	**Moscow #23**	**Moscow #26**	**H3R04P23**	**H3R04P28**	**H3R04P30**	**H3R13P40**	**OSU 1185.126**	**OSU 533.029**	**Ogyoo**	**Estrella #1**

**No. seedlings**	**32**	**32**	**46**	**46**	**46**	**46**	**32**	**46**	**32**	**32**
LG6 marker										
GB310	–	0.68	–	–	–	–	–	–	–	–
GB456	–	–	–	–	–	–	–	–0.87	–	
GB478	–0.52	–	–	–	–	–	–	–	–	
GB871	–	–	–	–	–	–	–	–0.93	–	
GB917	–	–	–	–	–	–	–	–0.93	0.67	
GK6.61	–	–	–	–	–	–	–	–	1.00	0.59
GK6.63	0.68	0.68	0.83	0.86	0.57	0.91	–	0.63	1.00	–
GK6.77	0.96	–	–	–	–	–	–	–	1.00	0.61
GK6.81	–	–	0.87	0.95	0.51	-	–	0.69	1.00	0.67
GK6.89	0.76	–	–	–	–	–	–	–	1.00	0.66
GK6.90	–	–	–	–	–	–	0.88	–	1.00	–
GK6.92	0.76	–	–	–	–	–	0.87	–	1.00	0.61
GK6.94	–	–	–	–	–	–	–	–	1.00	0.58
LG610	–	–	–	–	–	–	–	–	–	0.64
LG628	–	–	–	–	–	–	–	–	–	0.55
LG631	–	–	–	–	–	–	0.87	–	0.93	0.6
LG637	–	–	–	–	–	–	0.93	–	1.00	0.61
LG639	–	0.75	–	–	–	–	0.87	–	–	0.66
LG648	0.76	0.81	–	–	–	–	0.93	–	–	–
LG668	–	–	–	–	–	–	0.87	–	–	–
LG682	0.75	0.81	0.81	0.38	0.49	0.86	0.87	0.64	1.00	0.62
LG687	–	0.74	–	–	–	–	–	–	–	–
LG688	–	–	–	–	–	–	0.87	–	1.00	0.60
LG2 marker										
B509	–	–	–	–	–	–	–	–	–	–
B758	–	–	0.21	−	−	0.17	–	−	–	–
MS0026.08	–	–	0.13	0.16	0.08	0.11	–	0.22	–	–
MS0026.10	–	–	–	–	–	–	–	–	–	–
MS0026.14	–	–	0.14	0.13	0.09	0.15	–	0.16	–	–
MS0107.02	–	–	–	–	–	–	–	–	–	–
MS0113.04	–	–	0.21	0.01	−	0.13	–	0.12	–	–
MS0140.02	–	–	–	–	–	–	–	–	–	–
LG7 marker										
B020	–	–	–	–	–	–	–	–	–	–
B751	–	–	–	–	–	–	–	–	–	–
B753	–	–	–	–	–	–	–	–	–	–
KG817	–	–	–	–	–	–	–	–	–	–
MS0046.02	–	–	−	0.22	0.03	−	–	0.61	–	–
MS0046.07	–	–	–	–	–	–	–	–	–	–
MS0061.02	–	–	0.04	0.03	0.01	0.11	–	0.56	–	–
MS0076.08	–	–	–	–	–	–	–	–	–	–
MS0170.02	–	–	−	−	−	0.06	–	0.49	–	–

**TABLE 6:** Correlation coefficients for disease and simple sequence repeat marker scores in four hazelnut progenies segregating for resistance to eastern filbert blight.

**Progeny**		**12032**	**11027**	**11032**	**11029**

**Resistance source**		**Moscow #37**	**Winkler**	**OSU 366.060**	**Bauman**

**Resistance origin**		**Moscow**	***C. americana***	***C. americana***	***C. americana***
No. seedlings		32	32	32	32
Linkage group	Marker	–	–	–	–
6	GK6.81	–	0.05	0.16	–
6	LG628	–	0.01	–	–
6	LG631	0.15	–	–	0.15
6	LG688	–	0.01	0.14	–
2	B509	–	–	0.20	0.12
2	MS26.0008	–	0.16	–	–
2	MS0026.10	0.21	–	–	—
2	MS0107.02	–	–	0.06	0.13
2	MS0113.04	0.19	–	–	0.05
7	B020	–	–	0.17	–
7	B751	–	0.02	–	–
7	B753	–	0.03	–	0.13
7	KG817	0.04	–	–	–
7	MS0046.02	0.07	–	–	–
7	MS0076.08	–	–	0.20	–
7	MS0170.02	–	–	0.18	–

*The markers are on three linkage groups. Low values indicate independence of disease and marker scores.*

### Mapping EFB Resistance From Eight Sources

Previously mapped and new SSR markers were used for construction of linkage maps for the eight EFB resistance sources on LG6. The initial maps were based on the marker and disease response scores recorded in a spreadsheet. Inspection identified very few conflicts between the score for disease response and for presence of adjacent SSR markers. A second version of the map was created after rescoring questionable disease scores as “unknown.”

**FIGURE 3:**
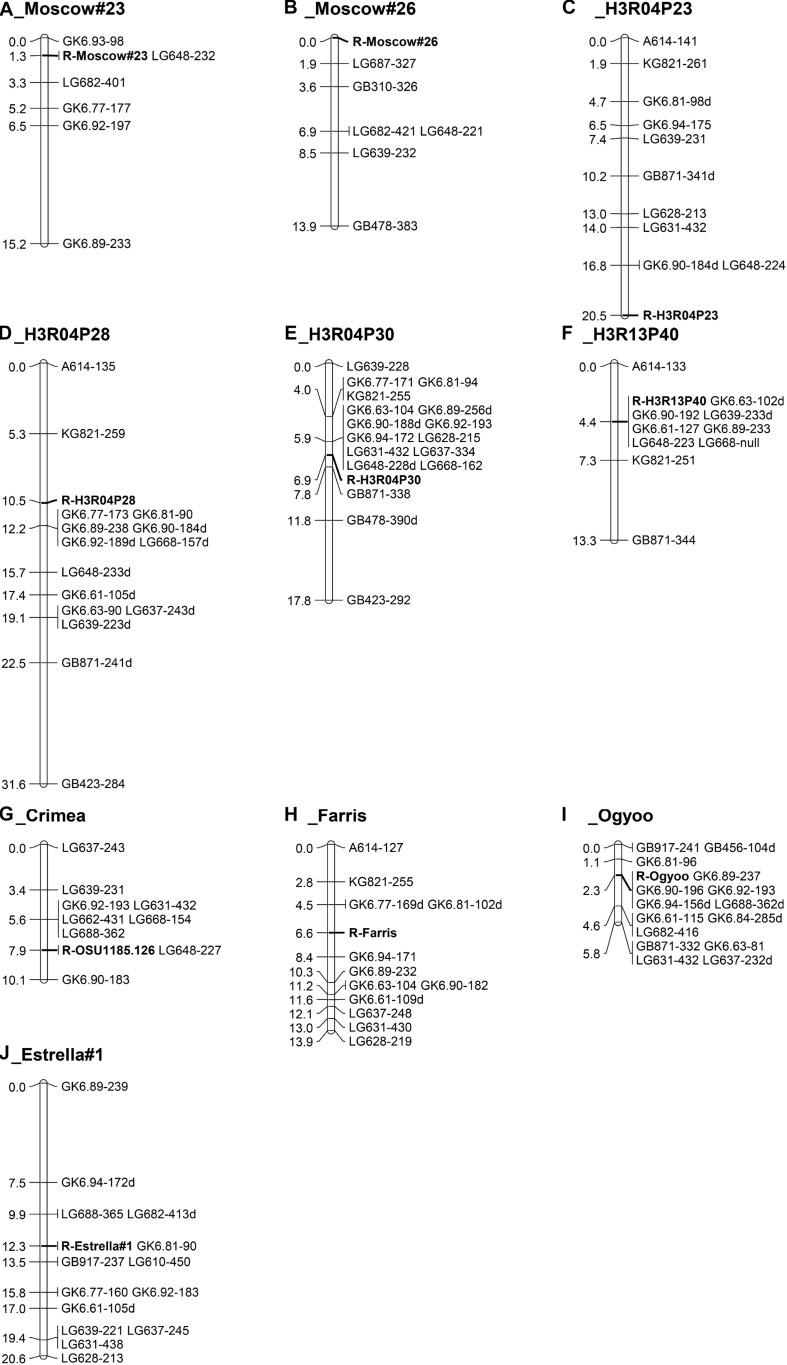
Maps of linkage group 6 (LG6) in hazelnut populations segregating for eastern filbert blight resistance from ten parents. **(A)** progeny 12028 for resistance from Moscow #23, **(B)** progeny 12029 for resistance from Moscow #26, **(C)** two progenies for resistance from H3R04P23 (RUS-13, Holmskij, Russia), **(D)** progeny 14029 for resistance from H3R04P28 (RUS-13), **(E)** progeny 14030 for resistance from H3R04P30 (RUS-13), **(F)** progeny 14036 for resistance from H3R04P30 (RUS-9, Holmskij, Russia), **(G)** progeny 11025 for resistance from OSU 1185.126 (RUS-26, Simferopol, Crimea), **(H)** two progenies for resistance from Farris OSU 533.029, **(I)** progeny 10021 for resistance from *C. heterophylla* ’Ogyoo’, and **(J)** progeny 11520 for resistance from ’Estrella #1’.

The map for progeny 12028 (Figure 3A, *n* = 78), which segregates for resistance from Moscow #23, was drawn with six markers, and shows resistance co-segregating with LG648 and flanked by markers GK6.63 and LG682 at distances of 1.3 cM and 2.0 cM, respectively. The map for progeny 12029 (Figure 3B, *n* = 65), which segregates for resistance from Moscow #26, has 6 markers and spans 13.9 cM with the resistance locus at the end and markers LG687 and GB310 placed 1.9 and 3.6 cM away. Three selections (H3R04P23, H3R04P28 and H3R04P30) were selected from seed lot RUS-13 from Holmskij, Russia. H3R04P23 is a parent of progenies 14027 and 14028. Initially a separate map was drawn for each progeny, and then a single map (Figure 3C, *n* = 111) was drawn for the merged data. Resistant selections H3R04P28 and H3R04P30 are the parents of progenies 14029 and 14030, respectively, and the maps are presented (Figure 3D, *n* = 61; Figure 3E, *n* = 53). In the two progenies segregating for resistance from H3R13P40, selected from seed lot RUS-9, also from Holmskij, Russia, resistance co-segregated with seven markers and was flanked by markers A614 and KG821 at distances of 4.4 and 2.9 cM, respectively (Figure 3F, *n* = 78). The map for progeny 11025, which segregates for resistance from OSU 1185.126 (selected from a seed lot from Crimea), was drawn with nine markers. The map (Figure 3G, *n* = 95) spans 10.1 cM with LG648 co-segregating with resistance and flanking markers on both sides.

The two progenies (14023 and 14024) segregating for resistance from Farris OSU 533.029 showed a surplus of seedlings scored as resistant (Table 2), although in progeny 14023 the correlation coefficients with markers on LG6 were > 0.62. Alignment of disease and LG6 marker scores in progeny 14023 indicated that 10 of the 54 seedlings were likely “escapes”. In progeny 14024, 9 of the 56 seedlings appear to be “escapes” and two seedlings with intermediate disease ratings of 3 had marker alleles indicating resistance. The disease scores in these seedlings were recoded as “u” and the maps redrawn. After recoding the disease response in these 22 seedlings, a map was drawn from the merged data of the two progenies. This map (Figure 3H, *n* = 110) shows resistance from Farris OSU 533.029 in the middle, with four markers on one side and eight on the other side. After the disease responses of the seedlings were reassessed based on marker scores, a total of 19 escapes were reclassified as susceptible and three seedlings with small cankers but markers for resistance were reclassified as resistant. In contrast to the original segregation ratios (Table 2), the revised ratio of resistant to susceptible seedlings is 28:26 in progeny 14023, 33:23 in progeny 14024, and 61:49 overall, with all of these ratios fitting the expected 1:1 ratio. We conclude that resistance from Farris OSU 533.029 is conferred by a dominant allele at a single locus on LG6 but that several seedlings escaped infection in this structure inoculation.

The map for progeny 10021 (Figure 3I, *n* = 88), which segregates for resistance from *C. heterophylla* ’Ogyoo’, includes 15 SSR markers and spans 5.8 cM. Five markers co-segregate with resistance, and additional markers flank the resistance locus on both sides. Progenies 11520 and 11521 segregated for resistance from ‘Estrella #1’ and segregation ratios from structure exposure showed good fit to the 1:1 expected ratio. In the field, dwarf seedlings were noted in both progenies. In progeny 11520, seven weak seedlings died when they were very young, two others were severely stunted, and 53 were of normal size. In progeny 11521, four weak seedlings died when they were very young, 13 others were dwarfs, and 45 were of normal size. After removal of these weak seedlings, the segregation ratios in both progenies fit the1:1 expected ratio. Furthermore, the homogeneity chi-square values indicated that it was appropriate to pool the data for progenies 11520 and 11521 in both structure and field exposure, and all fit the 1:1 expected ratio. The map for progeny 11520 (Figure 3J, *n* = 88) includes 14 SSR markers and spans 20.6 cM, with marker GK6.81 co-segregating with resistance, LG688 and LG682 on one side and LG610 and GB917 on the other side. Inspection of marker scores revealed the same segregation seen for resistance, with a slight deficiency of SSR alleles linked to resistance in the seedlings.

In the progenies investigated by field exposure, a total of nine individuals were recorded as a resistant while the SSR data indicated susceptibility, and it is likely that these seedlings escaped infection from *A. anomala* by chance. An additional four seedlings were scored as susceptible but their SSR data indicated resistance. As noted earlier, disease scores for these seedlings were recoded as “unknown” before the final maps were drawn.

## Discussion

The ‘Jefferson’ genome sequence (V2) allowed efficient development of new SSR markers. The V2 sequence (Snelling et al., 2018) was from Pacific Biosciences reads, for which the V1 Illumina sequences (Rowley et al., 2018) were used for error correction. SSRs continue to be widely used in plant genetics. Polymorphic SSRs often segregate in many different progenies, map to a single point in the genome, and serve as anchor loci for the alignment of multiple linkage maps. In this study, 42 new polymorphic SSR markers were developed from four contigs in the ‘Jefferson’ genome sequence (V2), and 19 were placed on the reference linkage map. Markers developed from contigs 95F and 77F mapped to LG6 as expected, but markers developed from contigs 56F and 42F mapped to LG1. This was unexpected, as the physical map of the EFB resistance region in ‘Jefferson’ (Sathuvalli et al., 2017) include the BACs 53B4 and 78A4, which in this study identified contigs 42F and 56F, respectively. It is likely that these two BACs were false positives in the BAC library screening. Unexpected LG assignments were also reported by Sathuvalli and Mehlenbacher (2013), including SSR markers LG612 and LG613 developed from BAC 38N24, which mapped to LG1. Additional false positives in their study were LG605 developed from BAC 62A9, which mapped to LG4, and LG655 and LG657 from BAC 65G23, which mapped to LG5. SSR markers developed for hazelnut are transferable across *Corylus* species (Bassil et al., 2005a, 2013, Boccacci et al., 2005) and even the related genera *Betula* and *Alnus* in the family Betulaceae (Gürcan and Mehlenbacher, 2010b). Future research, including saturation of the LG6 map with single nucleotide polymorphism markers, and alignment of these markers and BAC end sequences with the V3 ‘Jefferson’ genome sequence will allow rapid identification of genes of interest. The V3 ‘Jefferson’ genome sequence is from PacBio sequencing plus Hi-C proximity ligation (Dovetail Genomics, Scotts Valley, CA, United States) and consists of 11 scaffolds that represent the haploid number of hazelnut.

Segregation for response to EFB inoculation was studied in 18 progenies from 14 resistant parents representing 12 resistance sources (Table 2) of diverse origins. Seven sources are of Russian origin (Moscow #23, Moscow #26, Moscow #37, and four selections from seeds purchased in Holmskij), one (OSU 1185.126) originated from seeds purchased near Simferopol, Crimea, one (OSU 533.029) is from a seed lot received from Michigan, United States, and two are hybrids with other *Corylus* species. ‘Ogyoo’ (HF13) was selected from wild *C. heterophylla* in the South Korea. ’Estrella #1’, an interspecific hybrid from *C. sutchuenensis* × *C. avellana* ‘Holder’ (Farris, 1974), showed complete resistance to EFB after greenhouse inoculation (Chen et al., 2007) and 6 years of exposure in a field planting with high disease pressure (Capik and Molnar, 2012). Farris (1974) grew out several dozen seedlings from the cross and they appeared to be true hybrids based on morphology, but some were dwarf and stunted while others were vigorous and healthy. He selected the five best plants and named them Estrella hybrids #1 to #5. The observation of weak seedlings in the offspring of ‘Estrella #1’ in this study is consistent with the breeder’s notes for the original cross. ‘Estrella #1’ yields well and produces medium-size nuts with a slightly long shape but is male-sterile. The phenotype of Bauman selection OSU 401.014 indicates that it is a hybrid of *C. americana* × *C. avellana*, which would not be surprising as the hazelnut collections of many members of the Northern Nut Growers Association include both parent species and interspecific hybrids. In progenies segregating for resistance from 12 of these 14 sources, the segregation ratio for disease response fit the 1:1 ratio expected for control by single loci at which the dominant alleles confer resistance, and the resistant parent is heterozygous. The two progenies segregating for resistance from Farris OSU 533.029 had an excess of seedlings lacking disease and SSR markers indicated that a high number of seedlings had escaped infection. When these escapes were reclassified, the segregation ratios fit the expected 1:1 ratio. The one progeny segregating for resistance from ‘Moscow #37’ had an excess of susceptible seedlings. An excess of susceptible seedlings was reported in previous EFB resistance studies in hazelnut (Colburn et al., 2015; Bhattarai et al., 2017b). Lunde et al. (2006) noted an excess of resistant seedlings in the offspring of ’Zimmerman’, and that even when SSR markers indicate that the resistance gene is present, small cankers occasionally develop. Correlation with scores for alleles at mapped SSR markers allowed resistance from ten sources to be assigned to LG6 in the same region as ‘Gasaway’ resistance, and linkage maps were constructed and compared using common SSR markers. Disease response in progenies segregating for the remaining four resistance sources (Moscow #37, *C. americana* ‘Winkler’ and OSU 366.060, and Bauman hybrid OSU 401.014) was not correlated with scores for markers on LG6 or LG7 or LG2. The resistance from these sources might be due to a major gene on one of the other 8 LGs, and will be investigated further. Resistance on different linkage groups will be especially useful for R-gene pyramiding.

Most resistance genes follow the gene-for-gene hypothesis (Flor, 1956), which states that for every resistance gene in the plant, there is a corresponding *Avr* gene in the pathogen that confers avirulence. The host’s resistance gene allows it to detect and defend against the invader. Of the categories of resistance genes, those with a nucleotide binding site and leucine rich repeat (NBS-LRR) are most common, are associated with resistance to several plant pathogens, and are the targets of many investigations (Hulbert et al., 2001; Morata and Puigdomènech, 2017). Most NBS-LRR genes are physically clustered in plant genomes (Meyers et al., 2003; Zhou et al., 2004), possibly the result of duplication or amplification of the gene families. ‘Jefferson’ carries resistance inherited from ‘Gasaway’ and is heterozygous at a resistance locus on LG6. Sathuvalli et al. (2017) performed fine mapping of the region and suggested candidates for the EFB resistance gene. Further research is needed to determine if the ten new sources of resistance on LG6 investigated in this study are the same gene as ‘Gasaway’ or different genes.

Several studies have described the pyramiding of major genes for disease resistance as an approach for more durable resistance (Joshi and Nayak, 2010; Zhu et al., 2012), a strategy that would be facilitated by molecular markers tightly linked to the different resistance alleles (Mundt, 2018). The new SSR markers developed in this study and placed on LG6 near the ‘Gasaway’ resistance locus will be useful in marker-assisted selection and cultivar fingerprinting. Of these, the most promising from contig 77F are KG6.63 and GK6.61 and from contig 95F are GK6.81 and GK6.92 as they have high PIC values, few null alleles, and are easy to score. As breeder-friendly markers, they are alternatives to the RAPD markers 152-800 and 268-580 currently used for MAS. All of the new sources of resistance to *A. anomala* investigated in this study are promising for use in breeding. Eight sources were assigned to LG6, for which the newly developed SSR markers will aid the pyramiding of resistance genes. Four additional sources are also useful for breeding but their resistance has not yet been mapped.

## Data Availability Statement

The datasets presented in this study can be found in online repositories. The names of the repository/repositories and accession number(s) can be found below: https://www.ncbi.nlm.nih.gov/genbank/, MK913590-MK913608 and MT670379-MT670401. Marker sequences are available from GenBank.

## Author Contributions

SM conceived, designed, and secured funding for the study and wrote the first draft of the manuscript. GK developed, characterized, and mapped the new markers. GK and MŞ studied segregation for disease response, correlation of disease response and markers, mapping of resistance regions, and statistical analysis. JS assisted in marker development and scoring. GK, MŞ, and JS wrote sections of the manuscript. All authors contributed to manuscript revision, read, and approved the submitted version.

## Conflict of Interest

The authors declare that the research was conducted in the absence of any commercial or financial relationships that could be construed as a potential conflict of interest.

## Footnotes

1 http://www.fao.org/faostat/en

2 https://www.nass.usda.gov/Data_and_Statistics/index.php

3 http://oregonstate.edu/dept/botany/epp/EFB/index.htm

4 https://blast.ncbi.nlm.nih.gov/Blast.cgi

## Supplementary Material

Supplementary Material 1 Pedigrees of hazelnut (*Corylus avellana*) progenies segregating for resistance to eastern filbert blight. Resistant selections are underlined; all others are susceptible.

Supplementary Material 2 Primer sequences, linkage group assignments, and coordinates in the ‘Jefferson’ hazelnut genome sequence of 42 new simple sequence repeat markers in hazelnut.

Supplementary Material 3 Segregation and linkage group assignments of 19 simple sequence repeat markers in the reference hazelnut mapping population (OSU 252.146 × OSU 414.062).

## Abbreviations

EFB: eastern filbert blight
OSU: Oregon State University
SSR: simple sequence repeat.
