# Supplementary material for: New Sources of Eastern Filbert Blight Resistance and Simple Sequence Repeat Markers on Linkage Group 6 in Hazelnut (Corylus avellana L.)
Source: Front Plant Sci. 2021 Jun 14;12:684122. doi: 10.3389/fpls.2021.684122 (PMC8238048; doi:10.3389/fpls.2021.684122)
Supplement: Supplementary Material 1 — Pedigrees of hazelnut (Corylus avellana) progenies segregating for resistance to eastern filbert blight. Resistant selections are underlined; all others are susceptible. [file Data_Sheet_1.PDF]

Supplemental Material 1. Pedigrees of hazelnut (*Corylus avellana*) progenies segregating for resistance to eastern filbert blight. Resistant selections are underlined; all others are susceptible.

10021 = OSU 1181.002 x OSU 1093.107
OSU 1181.002 = OSU 731.112 x OSU 720.093
OSU 731.112 = OSU 373.095 x OSU 244.001
OSU 373.095 = *C. heterophylla* 'Ogyoo' x OSU 55.129
OSU 55.129 = Tonda Gentile delle Langhe x Tombul (Extra Ghiaghli)
OSU 720.093 = OSU 179.043 x OSU 313.078
OSU 179.043 = Henneman #3 x OSU 44.114
OSU 44.114 = Montebello x Compton
OSU 313.078 = OSU 23.017 x Tonda Gentile delle Langhe
OSU 23.017 = Barcelona x Tombul (Extra Ghiaghli)
OSU 1093.107 = OSU 746.092 x Ganja
OSU 746.092 = Tonda Pacifica x OSU 275.031
OSU 275.031 = Montebello x OSU 74.37
OSU 74.037 = 14.084 x OSU 17.068
OSU 14.084 = Barcelona x Daviana
OSU 17.068 = Barcelona x Tombul Ghiaghli

11025 = OSU 1185.126 x OSU 856.064
OSU 1185.126 from seeds (RUS-26) purchased near Simferopol, Crimea
OSU 856.064 = OSU 539.044 x Tonda Pacifica
OSU 539.044 = OSU 275.031 x Sant Pere
OSU 275.031 (see progeny 10021)

11027 = OSU 1197.113 x OSU 1172.001
OSU 1197.113 = OSU 786.091 x OSU 577.060
OSU 786.091 = OSU 256.005 x OSU 439.063
OSU 256.005 = OSU 54.046 x OSU 17.083
OSU 54.046 from seeds collected in Giresun, Turkey by M.M. Thompson
OSU 17.083 = Barcelona x Camponica
OSU 439.063 = Ribet x Willamette
OSU 577.060 = Tonda di Giffoni x Clark
OSU 1172.001 = OSU 527.017 x OSU 581.039
OSU 527.017 = *C. americana* 'Winkler' x *C. avellana* pollen mixture
OSU 581.039 = OSU 312.030 x OSU 278.121
OSU 312.030 = OSU 23.017 x Tonda Romana
OSU 23.017 = Barcelona x Tombul (Extra Ghiaghli)

11029 = OSU 889.084 x OSU 1155.009
OSU 889.084 = OSU 401.014 x Tonda Pacifica
OSU 401.014 from seeds labeled *C. americana* from Ken Bauman in New Carlisle, Ohio
OSU 1155.009 = OSU 474.084 x OSU 540.084
OSU 474.084 = Lewis x Tonda di Giffoni
OSU 540.084 = OSU 43.091 x Sant Pere
OSU 43.091 = Montebello x Self (or Unknown)

Supplemental Material 1. Pedigrees of hazelnut (*Corylus avellana*) progenies (cont'd).

11032 = OSU 955.028 x OSU 1213.088
OSU 955.028 = *C. americana* OSU 366.060 x Lewis
OSU 1213.088 = OSU 786.091 x OSU 540.084
OSU 786.091 =OSU 256.005 x OSU 439.063
OSU 265.005 = OSU 54.046 x OSU 17.083
OSU 54.046 from seeds collected in Giresun, Turkey by M.M. Thompson
OSU 17.083 = Barcelona x Camponica
OSU 540.084 (see progeny 11029)

11520 = Estrella #1 x OSU 1174.033
Estrella #1 = *C. heterophylla* var. *sutchuensis* x *C. avellana* 'Holder'
OSU 1174.033 = Sacajawea x OSU 681.085
OSU 681.085 from seeds collected in Moscow, Russia by J. Henkin

11521 = Estrella #1 x OSU 1219.032
Estrella #1 (see progeny 11520)
OSU 1219.032 = OSU 687.023 x OSU 665.123
OSU 687.023 from seeds collected near Trabzon, Turkey

12028 = OSU 1266.005 x Moscow #23
OSU 1266.005 = OSU 539.031 x OSU 474.013
OSU 539.031 = OSU 275.031 x Sant Pere
OSU 275.031 (see progeny 10021)
OSU 474.013 = OSU 237.158 x OSU 55.129
OSU 237.158 = OSU 14.019 x Tonda Romana
OSU 14.019 = Barcelona x Butler
OSU 55.129 = Tonda Gentile delle Langhe x Tombul (Extra Ghiaghli)

12029 = OSU 919.031 x Moscow #26
OSU 919.031 = OSU 539.031 x OSU 474.013
OSU 539.031 (see progeny 12028)
OSU 474.013 (see progeny 12028)

12032 = Moscow #37 x OSU 1218.068
OSU 1218.068 = OSU 540.084 x OSU 616.018
OSU 540.084 (see progeny 11029)
OSU 616.018 = Tonda di Giffoni x OSU 252.146
OSU 252.146 = OSU 41.083 x OSU 17.028
OSU 41.083 = Montebello x Compton
OSU 17.028 = Barcelona x Tombul Ghiaghli

14023 = OSU 1390.008 x OSU 919.031
OSU 1390.008 = OSU 753.054 x OSU 533.029 (Farris)
OSU 753.054 = Iannusa Racinante x OSU 350.089
OSU 350.089 = Tombul Ghiaghli x Tonda Romana
OSU 919.031 (see progeny 12029)

Supplemental Material 1. Pedigrees of hazelnut (*Corylus avellana*) progenies (cont'd).

14024 = OSU 1390.008 x OSU 1322.038
OSU 1390.008 (see progeny 14023)
OSU 1322.038 = OSU 896.082 x OSU 620.032
OSU 896.082 = Nocchiolino Sangrato x OSU 443.107
OSU 443.107 = OSU 183.060 x OSU 54.056
OSU 183.060 = Montebello x OSU 14.084
OSU 14.084 = Barcelona x Daviana
OSU 54.056 from seeds collected in Giresun, Turkey by M.M. Thompson
OSU 620.032 = OSU 332.097 x OSU 313.078 (see progeny 10021)
OSU 332.097 = Montebello x OSU 74.037 (see progeny 10021)

14027 = H3R04P23 x OSU 978.058
H3R04P23 from seeds (RUS-13) purchased in Holmskij, Russia (near Krasnodar)
OSU 978.058 = OSU 581.034 x OSU 620.032 (see progeny 14024)
OSU 581.034 = OSU 312.030 x Clark
OSU 312.030 (see progeny 11027)

14028 = H3R04P23 x OSU 1378.046
OSU 1378.046 = OSU 943.020 x OSU 885.056
OSU 943.020 = OSU 617.015 x OSU 495.045
OSU 617.015 = OSU 311.046 x Clark
OSU 311.046 = OSU 23.017 x Tonda Gentile delle Langhe
OSU 23.017 = Barcelona x Tombul (Extra Ghiaghli)
OSU 495.045 from seeds received from southern Russia
OSU 885.056 = OSU 539.044 x Tonda Pacifica
OSU 539.044 = OSU 275.031 x Sant Pere
OSU 275.031 (see progeny 10021)

14029 = Sacajawea x H3R04P28
H3R13P40 from seeds (RUS-13) purchased in Holmskij, Russia (near Krasnodar)

14030 = Sacajawea x H3R04P30
H3R13P40 from seeds (RUS-13) purchased in Holmskij, Russia (near Krasnodar)

14036 = H3R13P40 x OSU 978.058
H3R13P40 from seeds (RUS-9) purchased in Holmskij, Russia (near Krasnodar)
OSU 978.058 (see progeny 14027)

14037 = H3R13P40 x OSU 1078.043
OSU 1078.046 (see progeny 14028)
