# Supplementary material for: New Sources of Eastern Filbert Blight Resistance and Simple Sequence Repeat Markers on Linkage Group 6 in Hazelnut (Corylus avellana L.)
Source: Front Plant Sci. 2021 Jun 14;12:684122. doi: 10.3389/fpls.2021.684122 (PMC8238048; doi:10.3389/fpls.2021.684122)
Supplement: Supplementary Material 2 — Primer sequences, linkage group assignments, and coordinates in the ‘Jefferson’ hazelnut genome sequence of 42 new simple sequence repeat markers in hazelnut. [file Data_Sheet_2.PDF]

Supplemental Material 2. Primer sequences, linkage group assignments, and coordinates in the 'Jefferson' hazelnut genome sequence (V3) of 42 polymorphic simple sequence repeat markers.

		Primer sequences (5--3')			Coordinates (V3)		
Marker	LG	Forward	Reverse	Allele and linkage group	Scaffold	Begin	End
GK1.05	1	Hex-TACAGGTCTGAAACAACGAAGC	ACCGCAAGTCAAGAGAGAAAAC	125 on 1F, 129 on 1M	2	30545974	30546591
GK1.09	1	Hex-TTTCCCTAAAATCTTCCCACG	AAAGAAATCAGAAGTGATCGCC	--	2	30828070	30828664
GK1.10	1	Fam-TGATTGCTCTCTCTCTCTTTGA	CTCGTATAAGCTCGTCTCGTACT	292 on 1F	2	30837848	30838471
GK1.12	1	Hex-TCAATGTTTTGAGGCAAGAGG	TGGACACTTTGGCATATTTGAG	124 on 1F	2	29182051	29182676
GK1.17	1	Fam-GCAGCCAGATGAGAGTGAAAG	CCATTGTGGATGATACCTCCTTA	--	2	31000174	31000793
GK1.18	1	Hex-GTTGAAACTGCTCCACGACAT	TTAGAAGATAAGCGGATCGAGG	--	2	31034638	31035253
GK1.20	1	Fam-TCCACCCACCTCTCCTATAAAT	CAAGCTCAAAATCTCCTCCTTC	152 on 1F	2	29431038	29431655
GK1.21	1	Fam-GAAATTGCGAGGTCCAGC	CACCCAAAACCTTCTCTCTCTC	--	2	29576880	29577476
GK1.24	1	Hex-CCATTCCAAAACACACTGCTAA	TGGCACTTCCTCCCTTTGTA	--	2	29909704	29910315
GK1.25	1	Fam-AGGGGTAGAAAGAAGAGCAAGG	AACTGGACTGAGCAAAGTGGTT	--	2	29931680	29932315
GK1.30	1	Hex-CCTCCACATGCCACTGCT	GCTGCAACCATAGTCCATAGAATTA	199 on 1F, 185 on 1M	2	58729956	58730589
GK1.35	1	Fam-TCCTCATGTCCCGGTGTC	TCACGACGACAACTCCCAG	--	2	60024574	60025192
GK1.36	1	Hex-CAGTTATTGGCATGGCTGG	GAGAGCGTGTAGCATTTCTCAA	--	2	60079631	60080236
GK1.38	1	Fam-ATCATACATGGTGGTGCCAATA	CATCAACAAGCCAATCACAACT	135 on 1M	2	60272233	60272864
GK1.39	1	Hex-TTGCAGAAATACGAGAGAGAGAGA	CAAAACCACCCAGTCCAGAT	--	2	57990672	57991305
GK1.40	1	Hex-TGTCATCTCCTTGCTGATGC	AACTAATTCCTCGGGTGGATG	369 on 1F, 367d on 1M	2	58886758	58887383
GK1.41	1	Fam-AGGCACGTTGGCTGTAGTTT	GGAAAATCCCAGACAGATGCTA	240d on 1F, 244 on 1M	2	58893862	58894489
GK1.43	1	Hex-ATGGAGAGGCTAACGAAGTCAT	CTGCCACGGAAGAAATATACAC	--	2	59182452	59183064
GK1.44	1	Hex-GCAATTACCACTCAAACTTGTCC	CATTGACATCTCGTCTCAGCAT	305 on 1F, 307 on 1M	2	59239614	59240243
GK1.45	1	Hex-AGCCACCCAGCCTCTTTT	CGTTTGAACAGTAAAATGCCTC	199 on 1F, 203 on 1M	2	59371268	59371891
GK1.46	1	Hex-GGACGGATTGCTTAAACCTAAA	GGTCAACCCTACCAAGAGAGAG	--	2	59432395	59433006
GK1.49	1	Fam-ATGGGTTGATCTTGGAGAAAG	CTTCTTTGTATGGGTTAGTACATCG	--	2	58856517	58857124
GK6.58	6	Fam-ATTCACGAGTCACCTTCTCTCC	AGTTGTGAGCAGTTTGGTTTTG	--	8	18439464	18440052
GK6.61	6	Fam-CTCATTCAAAGCACCCAACC	TCCAAATCACACAGCTCTTGTC	105 on 6F, 107 on 6M	8	20344184	20343561
GK6.62	6	Hex-AAGTTGCATTTCCGTCCAAT	TGCCAATAATCAGGAGAGTTCA	--	8	20323431	20324045
GK6.63	6	Fam-GCAAACTTCCAGAAAACCAA	AATGTTCGTAGGACAACTGCAT	101 on 6F, 79 on 6M	8	20280532	20279897
GK6.70	6	Hex-AGTGGGAACTCAGGGATATGCT	TTCTGCTTTTGTTCTTCCTGGT	--	8	19821765	19822379
GK6.71	6	Hex-GCCATGTCAACTCTATGCTATG	TTGGGCAAAGCTGAATCTAT	--	8	19750101	19750695
GK6.76	6	Hex-CAGAATGGTTGCTCCAATCC	CCATTTGGTGATTTTGATGC	--	8	19874160	19874751
GK6.77	6	Fam-GGGAAAAGCAAAAGAAATACATGG	ATCTCATAGGCCCAACAAACGC	177 on 6M	8	23659092	23659715

GK6.80	6	Hex-AAAAGAAATGGATGTGACCCAG	GACAAGAAAGAAGAGCCACCAC	184 on 6M	8	23858631	23859246
GK6.81	6	Fam-CCACACAGAGAAGAATATCGCA	GTTGTTGGTAAGGTCGGCTTC	100 on 6F, 98 on 6M	8	23883093	23883718
GK6.82	6	Hex-TTATTCCTATTCTTCCGCATGG	CTGGCACCAACATTCATAACAT	--	8	23883104	23883703
GK6.83	6	Fam-ACCTCACGCCCGTCTTCT	CGTTCTGGGGTGTTGTTTAGTG	--	8	21792280	21792891
GK6.84	6	Hex-GTGGCTAATCCCCTATCTCCTT	GTTGGAAAGACAGCTTGGACTT	297 on 6F	8	22852844	22853473
GK6.89	6	Hex-GAACAGGGGCAATTAGATGGTA	GCCTTTCTAGTGTTGTGCCAG	256 on 6F, 230 on 6M	8	23127189	23127812
GK6.90	6	Hex-AGTGGTGAGAGGGGCTACATAA	TATATTCAAACAGGTGCTGCGA	188 on 6F, 190 on 6M	8	23133341	23133964
GK6.91	6	Hex-CAAAACCTCTTCCCACCTTTTA	ACAGGAGCTTGTCTTGCGTAT	124 on 6M	8	23147261	23147858
GK6.92	6	Fam-ACCTTTCCCATAAACCACCTTT	TGGTACAAAGACAAAGAGGAGAGA	194 on 6F, 196 on 6M	8	23241826	23242455
GK6.94	6	Fam-CAAACATCTTCTCGTCCAATCA	TTAAATACCCCACTGCCCTTC	176 on 6F, 168 on 6M	8	23556149	23556772
GK6.95	6	Hex-AATTGTCAGAAGCCACCTCAAT	TGAATGAGAAACGTGGGAAGTA	--	8	23571735	23572350
GK6.97	6	Hex-CTTAACGTGTATCGGGTCTCCT	GAGAAGATCAAGTCGCTGGC	--	8	22792755	22793372
