# Supplementary material for: New Sources of Eastern Filbert Blight Resistance and Simple Sequence Repeat Markers on Linkage Group 6 in Hazelnut (Corylus avellana L.)
Source: Front Plant Sci. 2021 Jun 14;12:684122. doi: 10.3389/fpls.2021.684122 (PMC8238048; doi:10.3389/fpls.2021.684122)
Supplement: Supplementary Material 3 — Segregation and linkage group assignments of 19 simple sequence repeat markers in the reference hazelnut mapping population (OSU 252.146 × OSU 414.062). [file Data_Sheet_3.PDF]

Supplemental Material 3. Segregation and linkage group assignments of 19 simple sequence repeat marker loci in the hazelnut reference mapping population (OSU 252.146 x OSU 414.062).

Locus	Allele sizes in parents (female x male)	Observed ratio	Expected ratio	Chi-square df	Chi-square Value	Chi-square P
GK1.05	125/131 × 123/129	24:34:37:42	1:1:1:1	3	5.04	0.168
GK1.10	286/292 × 286/null	35:78:24	1:2:1	2	4.40	0.11
GK1.12	124/124 × 126/null	59:77	1:1	1	2.38	0.12
GK1.20	152/158 × 170/170	59:77	1:1	1	2.38	0.12
GK1.30	191/199 × 185/191	35:41:29:32	1:1:1:1	3	2.30	0.51
GK1.38	129/133 × 131/135	36:33:36:30	1:1:1:1	3	0.73	0.87
GK1.40	363/369 × 363/367	35:40:30:31	1:1:1:1	3	1.82	0.61
GK1.41	240/248 × 244/248	36:39:30:31	1:1:1:1	3	1.59	0.66
GK1.44	301/305 × 293/307	37:35:30:28	1:1:1:1	3	1.63	0.65
GK1.45	191/199 × 203/205	34:35:30:37	1:1:1:1	3	0.76	0.86
GK6.61	105/111 × 107/111	30:40:33:33	1:1:1:1	3	1.59	0.66
GK6.63	93/101 × 79/93	33:34:31:39	1:1:1:1	3	1.01	0.80
GK6.77	173/173 × 173/177	74:63	1:1	1	0.88	0.35
GK6.81	90/100 × 90/98	35:33:41:29	1:1:1:1	3	2.17	0.54
GK6.84	291/297 × 291/null	41:67:29	1:2:1	2	2.17	0.34
GK6.89	228/256 × 228/230	35:33:40:29	1:1:1:1	3	1.83	0.61
GK6.90	184/188 × 184/190	34:33:41:29	1:1:1:1	3	2.18	0.54
GK6.92	188/194 × 188/196	34:33:41:29	1:1:1:1	3	2.18	0.54
GK6.94	172/176 × 168/172	35:32:29:41	1:1:1:1	3	2.30	0.51
